# Has Large-Scale Named-Entity Network Analysis Been Resting on a Flawed Assumption?

**DOI:** 10.1371/journal.pone.0070299

**Published:** 2013-07-24

**Authors:** Brent D. Fegley, Vetle I. Torvik

**Affiliations:** 1 Graduate School of Library and Information Science, University of Illinois at Urbana-Champaign, Champaign, Illinois, United States of America; 2 Illinois Informatics Institute, University of Illinois at Urbana-Champaign, Urbana, Illinois, United States of America; Université de Lausanne, Switzerland

## Abstract

The assumption that a name uniquely identifies an entity introduces two types of errors: *splitting* treats one entity as two or more (because of name variants); *lumping* treats multiple entities as if they were one (because of shared names). Here we investigate the extent to which splitting and lumping affect commonly-used measures of large-scale named-entity networks within two disambiguated bibliographic datasets: one for co-author names in biomedicine (PubMed, 2003–2007); the other for co-inventor names in U.S. patents (USPTO, 2003–2007). In both cases, we find that splitting has relatively little effect, whereas lumping has a dramatic effect on network measures. For example, in the biomedical co-authorship network, lumping (based on last name and both initials) drives several measures down: the global clustering coefficient by a factor of 4 (from 0.265 to 0.066); degree assortativity by a factor of ∼13 (from 0.763 to 0.06); and average shortest path by a factor of 1.3 (from 5.9 to 4.5). These results can be explained in part by the fact that lumping artificially creates many intransitive relationships and high-degree vertices. This effect of lumping is much less dramatic but persists with measures that give less weight to high-degree vertices, such as the mean local clustering coefficient and log-based degree assortativity. Furthermore, the log-log distribution of collaborator counts follows a much straighter line (power law) with splitting and lumping errors than without, particularly at the low and the high counts. This suggests that part of the power law often observed for collaborator counts in science and technology reflects an artifact: name ambiguity.

## Introduction

Assuming that an author is uniquely identified by last name and first initials, Newman [1] reported much greater clustering and assortative mixing of co-authorship networks in physics versus biomedical science. This implied that physicists tended to collaborate much more transitively and homogenously than did biomedical scientists. Could this result be an artifact of a flawed assumption and simply reflect the extent of name ambiguity? After all, we know experientially that name ambiguity increases with the size and diversity of bibliographic databases and that the biomedical literature is much larger than that of physics.

Name ambiguity is not unknown to investigators who study large networks; but few attempt to resolve this bias because it requires substantial effort. Many argue that ambiguity has a small effect on statistical network measures (e.g., [2]–[7]). Some ignore the matter completely (e.g., [8]–[15]). However, some have found that measurement errors vary significantly depending on an author’s role. For example, measurements of individuals acting as network “bridges” or “hubs” are subject to the greatest systematic distortion [16].

**Figure 1:**
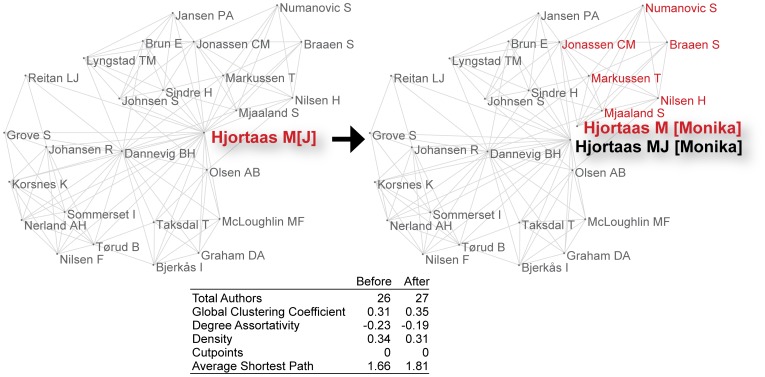
Illustration of splitting 1 author into 2 authors based on a name variant alone. The bold arrow separating the 2 network diagrams indicates the direction of change: before, to the left; after, to the right. Hjortaas M[J] is split into Hjortaas M and Hjortaas MJ based on last name, both initials. Note that the split would not have occurred if last name, first initial had been the criterion. Note also that the artificial vertices created by the split do not separate completely in the sense that Hjortaas M and Hjortaas MJ continue to share some co-authors. This is real data from PubMed; but the network measures regard the present, local network only.

**Figure 2:**
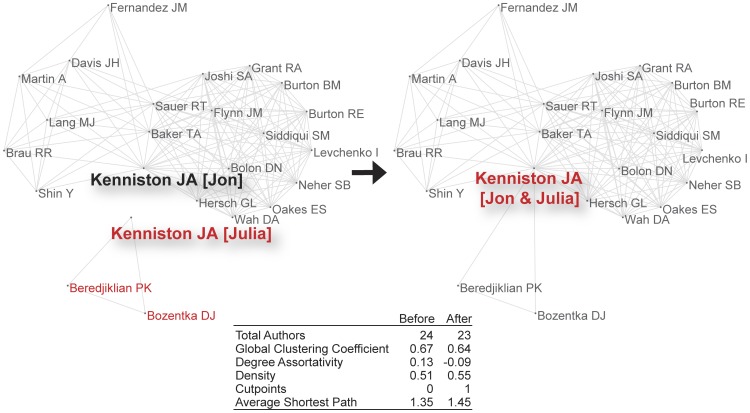
Illustration of lumping 2 authors using only last name, both initials. The bold arrow separating the 2 network diagrams indicates the direction of change: before, to the left; after, to the right. Note that Jon A. Kenniston and Julia A. Kenniston had no common co-authors before lumping. Lumping introduces a cutpoint as 2 connected components become biconnected. This is real data from PubMed; but the network measures regard the present, local network only.

Two types of errors result from assuming that a name identifies an individual uniquely: *splitting*, whereby output by one individual is assigned to two or more individuals (e.g., due to name variants); and *lumping*, whereby output by several individuals is assigned to one individual (e.g., due to common names). Figures 1 and 2 illustrate these two types of errors on co-authorship networks using data from the Author-ity 2009 dataset (a computational disambiguation of authors in PubMed; [17], [18]). Figure 1 shows two graphs before and after a splitting error. The vertex corresponding to Monika J. Hjortaas is erroneously split into two new vertices (labeled M. Hjortaas and M. J. Hjortaas) when we assume that individuals are identified by a name (here, last name, both initials). The two new vertices are connected to two different sets of co-authors that are *not* mutually exclusive (e.g., C. M. Jonassen appears in both sets, representing an indirect connection between the two new “authors”). In Figure 2, the vertices corresponding to Julia A. Kenniston and Jon A. Kenniston are erroneously lumped into one vertex when we assume that a name (here, last name, both initials) identifies the individual. Before lumping, the network comprises two disjoint subgraphs (one for Julia; the other for Jon); after lumping, the network comprises a pair of biconnected components sharing a single cutpoint, J. A. Kenniston. Splitting and lumping change network topology in these small examples, and the accompanying network measures reflect a modest distortion.

We hypothesize that the distortion of network measures becomes more pronounced in networks that are larger and more comprehensive. To test this hypothesis, we investigate the effects of splitting and lumping on different statistical properties of two large-scale networks: one based on PubMed co-authorship; the other on USPTO co-inventorship. Starting with bibliographic datasets where names have been computationally disambiguated, Author-ity 2009 [17], [18] and USPTO [19], we simulate splitting and lumping and show that these errors will indeed affect many network measures in dramatic, if not counterintuitive, ways.

### Disambiguation Efforts

Name ambiguity jeopardizes research, collaboration, decision-making, and commerce. To appreciate this fact, one need only consider large-scale initiatives that attempt to counteract ambiguity’s effects: *authority control* (which has a long history in library information systems management), the Virtual International Authority File (VIAF; http://www.oclc.org/viaf/), The Friend of a Friend (FOAF; http://www.foaf-project.org/) project, Master Data Management (MDM), OpenID (http://openid.net/), VIVO [20], and Open Researcher & Contributor ID (ORCID; http://www.orcid.org/), to name only a few. The problem these initiatives address – the lack of a definitive link between an individual or entity and its output or manifestations – permeates institutional, disciplinary and commercial repositories alike; and the responsibility for maintenance is not always clear. For example, to what extent can a depositor be trusted to find the correct identifier for a co-author let alone spend time in its pursuit [21]? Torvik and Smalheiser [18] found that MEDLINE is rife with instances of author name ambiguity, due partly to changing data curation practices over years. Full first names appeared for the first time in MEDLINE in the year 2002. Other identifying information such as affiliations and email addresses were added more recently, and their use has been inconsistent. (See also [22]–[24].).

Name ambiguity exists because it is both useful and necessary in reducing redundancy and enabling reuse. It limits the cognitive resources required to identify a person, place, or thing; and it makes language (natural or artificial) efficient [25]. The price paid is the time, energy, and care needed to simultaneously identify and differentiate entity instances and their referents accurately. Name variants resulting from Romanization, misspelling, use of abbreviations, aliases or nicknames, changes in marital status, and religious conversion present a particular challenge for disambiguation of human names [26]. Moreover, even when linguistically simple (as opposed to descriptive) proper names can be matched, disambiguation without contextual clues may distort the topology of the network itself, as Figures 1 and 2 illustrate. This distortion becomes acute when the sample under study contains a significant number of common surnames and given names. [21], [27].

Algorithmic name disambiguation is also known under names such as *entity resolution* and *coreference resolution* (in natural language processing), *record linkage* and *duplicate detection* (in administrative database management), among many others. The absence of standards for name disambiguation, lack of benchmark datasets, context-specificity, poor performance, and difficulty scaling algorithms are reasons offered for avoiding the arduous task of disambiguation altogether. Many features must be considered in combination to disambiguate with a high degree of accuracy. For example, co-author names, affiliations, self-citations or references, topics, vocabulary, temporal features, and name frequency all contribute non-redundant information in disambiguation [4], [16], [26], [28], [29]. Restricting the domain of inquiry (e.g., to information science or stem cell research) and even the byline position of authors, along with author co-citation analysis, has helped in the past too; but the increasing presence of common Chinese and Korean surnames (e.g., Liu, Chen, Kim, Lee) among authors complicates this particular approach [30]. Some investigators have offered methods to assess the cost of name disambiguation in natural language processing [31] and bibliographic analysis [16]. Others have taken steps to establish and improve benchmark datasets [32]–[35] and to encourage participation in improving matching algorithms through competition at an institutional level [36].

## Materials and Methods

### Network Measures

A network is defined as a set of vertices (or nodes), *V*, and a set of edges, *E*, each of which connects a pair of vertices. To facilitate comparison with other work (specifically [1], [37]), we treat edges as undirected and unweighted, even though we could define direction in terms of the position of an author on a publication and weight as the frequency of co-authorship, for example. Additionally, no two vertices may have more than one edge between them; and no vertex may be self-referential.

Several measures figure prominently in graph-theoretic analyses of collaboration networks: clustering coefficient, degree assortativity, and average shortest path length, among others. Each characterizes a different aspect of network topology. Several terms are rudimentary to this characterization. A *triple* (a.k.a. triad) is a graph or subgraph comprising 3 vertices that may or may not be interconnected. A *connected triple* has 2 or 3 edges. A connected triple with 2 edges is called a *2-star*; a connected triple with 3 edges is a *triangle*. Lastly, the *degree* of a given vertex is the number of edges incident to that vertex. (See [38]–[41]).

The word *collaboration* serves presently as shorthand for activity among individuals resulting in a published paper or a granted patent (co-authorship or co-inventorship, respectively). Co-authorship (or co-inventorship) is, in a sense, an approximation of collaboration and only captures certain types of collaboration, because (a) a paper is not sufficient evidence of collaboration among all pairs of authors on a paper [6], [42]; and (b) the lack of a co-authorship does not imply the absence of collaboration. Also, mere co-occurrence of authors does not capture the *nature* of their relationship.

The *clustering coefficient* of a collaboration network measures the degree to which people tend to collaborate transitively. A transitive relation is one where if vertex *X* directly connects to vertex Y, and Y directly connects to vertex *Z*, then *X* and *Z* are directly connected too. M. Granovetter [43], [44] famously explored transitive relations in the context of friendship networks, concluding that even if *X* and *Z* are not directly connected, they may indirectly integrate each other into disparate communities and increase opportunities for one another.

The clustering coefficient is calculated in two prominent ways differing on the unit of averaging: connected triples (Equation 1); or vertices (Equation 2; [45], [46]). For the former, the global clustering coefficient, *C,* is simply the proportion of connected triples in the network that are triangles (i.e., averaged over connected triples):
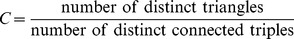
(1)


For the latter, one calculates a local clustering coefficient for each vertex and then computes the average over all vertices:
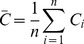
(2)


where *C_i_* is the clustering coefficient of the network formed by the *i*-th vertex and its neighbors; and *n* is the total number of vertices in the network. In both cases, the clustering coefficient ranges from 0 to 1, where 0 indicates that all vertices are arranged hierarchically (as trees or chains), and 1 indicates that all connected triples are triangles (where each friend of a friend is also a direct friend). Soffer & Vázquez [46] proposed an alternative definition of the clustering coefficient that factors out degree-degree correlations. Nevertheless, for the sake of comparison with results reported by [1], [37], we also use Equation 1.

*Assortativity* (a.k.a. assortative mixing, homophily) in a collaboration network is the extent to which authors collaborate with people like themselves. Here, degree is the unit of comparison. The greater the degree assortativity, the less vulnerable the network becomes to disruptions in information flow from targeted failure or removal of high-degree vertices [47], [48]. Newman [49] quantifies assortativity as “the Pearson correlation coefficient [*r*] of the degrees at either ends of an edge.” Pearson’s *r* ranges from −1 to 1. In the context of a collaboration network, if 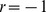, then participants collaborate exclusively with opposites; if 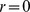, participants have no preference; if 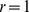, then participants collaborate exclusively with others like themselves. Kendall-Gibbons’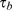 and Spearman’s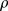, both rank correlations, are among proposed alternatives to Pearson’s *r*. [50]–[52].

*Density* is the proportion of vertex pairs that are directly connected. It has long been considered an indicator of structural (group) cohesion, because it measures the “completeness” of a network (that is, the extent to which all vertices connect to one another; [39], [53]). However, Friedkin [53] reiterates a caution that as an aggregate measure, density is not a reliable measure of structural cohesion at all if the network contains subgroups (e.g., groups with high local density in an otherwise low-density network). PubMed and USPTO most certainly contain subgroups; so among results, we also include the mean number of co-authors and co-inventors, respectively, in our tabulation of density. (See [39], p. 180ff).

A *connected component* is a maximal subgraph wherein each vertex is reachable from any other vertex. A *biconnected component* (a.k.a. block) is a connected component that joins another connected component via a *cutvertex* (a.k.a. cutpoint, cutnode, articulation point, or boundary spanner) or a *cutedge* (a.k.a. bridge or isthmus; [54]–[57]). *Cutpoints* and *bridges* are critical elements, because their removal creates connected components, eliminating one or more paths in the network and, consequently, disrupting the flow of information within the network.

The *shortest path* (a.k.a. geodesic distance) from one vertex to another is the path with the minimum number of edges. Knowing the shortest path between points has practical benefit when, for example, social navigation is critical for task completion. The breadth-first search algorithm finds the minimum paths between a starting vertex and all other vertices with time complexity 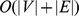 [57]. This measure is not tractable computationally for networks beyond a certain size; so we use sampling with negligible sampling error for estimates.

### Datasets

Author-ity 2009 is a set of inferred authors (∼9.3 million) from instances of author names on biomedical or biomedically-related publications (∼20 million) from ∼1946 to September, 2009 in PubMed. Author-ity 2009 is the product of an algorithm that clusters instances of author names associated with different PubMed records probabilistically and with a high degree of accuracy (∼98%; [17], [18]). The algorithm accomplishes this result by computing similarity profiles from many different fields in PubMed records. To maintain the window size used by [1], [37], we restricted papers (not exclusively journal articles) to those published in the 5 years between 2003 and 2007, inclusive. The papers within this range contain more complete metadata than earlier periods, helping remove any possible edge effects. For example, the majority of these papers include the given (first) names of authors; so accuracy should be better. To reiterate, previous assessment of the entire Author-ity dataset showed both splitting and lumping at less than 2% [17]; and the subset of Author-ity used in the present paper is likely to be more accurate, because papers are restricted to years which typically have more complete records (i.e., include first names and affiliations). Unless indicated otherwise, the names Author-ity 2009 and PubMed are interchangeable in this paper. An analogous 2003–2007 dataset was extracted from the USPTO (United States Patent and Trademark Office) inventor data disambiguated by [19].

Each dataset consists of a set of clusters, each of which contains a set of name instances on papers (or patents) representing an author (or inventor). Each cluster can have multiple name variants (making it eligible for splitting) and can share a name with other clusters (making it eligible for lumping).

### Simulations

Starting with a disambiguated dataset pruned of isolates (authors without any co-authors, inventors without any co-inventors), all possible splitting operations are performed followed by all possible lumping operations. Splitting (and lumping) operations in each simulation follow a random order. As illustrated in Figure 1, a splitting operation entails cleaving one randomly selected name variant (along with its name instances) from a randomly selected cluster to create two new clusters, at least one without any name variants. All splitting is complete when no cluster contains name variants. As illustrated in Figure 2, a lumping operation entails combining a pair of randomly selected clusters that share a name. Lumping is performed with two different definitions of name: last name, first initial; last name, both initials. All lumping is complete when no two clusters share a name. When all splitting and lumping is complete, each cluster is uniquely identified by a name (i.e., name = identity). In practice, we maintain a list of splitting and lumping eligibles to make random selection efficient and determine when to terminate the algorithm.

Each splitting or lumping operation creates a new network where a cluster corresponds to a vertex and the co-occurrence of a name instance on a paper (or patent) corresponds to an edge. All network measures are computed for each of 100 such networks sampled every *x* number of splitting (or lumping) operations, where *x* is the total number of operations divided by 100. Only the average shortest path is estimated by sampling, because of its computational complexity. Estimation involves randomly sampling 1,000 vertices from the giant component of each of the 100 networks. A second random sample confirmed that the sample size is sufficient to make sampling errors negligible. It should be noted that the networks obtained at the end of all lumping operations are equivalent to the networks studied in [1]–[15], [37] where it is assumed that a name uniquely identifies the person.

## Results

Tables 1–3 summarize the overall effects of splitting and lumping. They list measures for biomedical co-authorship networks (in PubMed) and co-inventorship networks (in USPTO) constructed in three different ways: one disambiguated; two non-disambiguated (based on two different definitions of a name). The two non-disambiguated networks correspond to the application of all splitting and lumping operations. In Table 1, the disambiguated PubMed and USPTO networks respond similarly to splitting and lumping errors as reflected by the direction of change in the value of all measures, except for the proportion of cutpoints. Among similarities, the following measures *decrease*: number of vertices (authors and inventors), number of biconnected components as a percentage of vertices, clustering coefficient, degree assortativity, and average shortest path. For example, for PubMed (from disambiguated to last name, both initials), the global clustering coefficient decreases by a factor of 4 (from 0.265 to 0.066); degree assortativity by a factor of ∼13 (from 0.763 to 0.06); and average shortest path by a factor of 1.3 (from 5.9 to 4.5). The following measures *increase*: mean degree (co-authors and co-inventors) and size of the giant component as a percentage of vertices. For PubMed (from disambiguated to last name, both initials), mean co-authors increases modestly by a factor of 1.5 (from 16.8 to 24.9); the size of the giant component as a percentage of vertices increases by 1.02 (95.5% to 97.4%). Stated another way, if one assumes that a name uniquely identifies an individual, then one will *underestimate* the number of individuals in the network, the degree to which individuals collaborate in a transitive fashion, and the extent to which individuals collaborate with others like themselves. Likewise, one will also *overestimate* an individual’s average number of collaborators and the extent to which the majority of individuals are connected. Also, the two different definitions of a name (last name, first initial, and last name, both initials) do not provide bounds on any of the measures, as Newman assumed [37].

**Table 1:** Extent of distortion caused by name = identity assumptions.

Network measures	Disambiguated	Last name, First initial	Last name, Both initials
**PubMed (2003–2007)**			
Authors	3.17×10^6^	1.56×10^6^	2.18×10^6^
Mean Co-authors	16.8 (40.9 SD)	32.4 (135.2 SD)	24.9 (94.4 SD)
Density	5.3×10^−6^	20.7×10^−6^	11.5×10^−6^
Giant Component	3.02×10^6^ (95.5%)	1.54×10^6^ (98.3%)	2.12×10^6^ (97.4%)
Biconnected Components	211,014 (6.7%)	72,295 (4.6%)	121,126 (5.6%)
Cutpoints	132,492 (4.2%)	52,003 (3.3%)	84,976 (3.9%)
Global Clustering Coefficient	0.265	0.046	0.066
Degree Assortativity	0.763	0.043	0.060
Average Shortest Path	5.88	3.99	4.49
**USPTO (2003–2007)**			
Inventors	468,697	258,221	344,755
Mean Co-inventors	4.6 (4.9 SD)	8.1 (23.4 SD)	6.4 (15.7 SD)
Density	9.9×10^−6^	31.5×10^−6^	18.5×10^−6^
Giant Component	214,195 (45.7%)	222,584 (86.2%)	254,080 (73.7%)
Biconnected Components	128,384 (27.4%)	53,582 (20.8%)	84,143 (24.4%)
Cutpoints	49,546 (10.6%)	27,835 (10.8%)	39,278 (11.4%)
Global Clustering Coefficient	0.268	0.031	0.043
Degree Assortativity	0.414	0.060	0.128
Average Shortest Path	12.95	5.04	6.35

The following table shows a variety of network measures for 2 different bibliographic databases (PubMed and USPTO) construed in 3 different ways. The label *Disambiguated* refers to baseline datasets generated by [17]-[19]; the other labels denote versions of these baselines derived using the given name = identity assumption. Note that the network measures vary considerably; those for global clustering coefficient and degree assortativity are particularly dramatic.

*Note:* SD = standard deviation.

**Table 2:** The effect of splitting and lumping on precision and recall for PubMed (2003-2007) and USPTO (2003-2007) with respect to disambiguated networks.

Network	Precision	Recall
**PubMed (2003–2007)**		
Split	89%	
Lumped, One initial		35%
Lumped, Both initials		40%
**USPTO (2003–2007)**		
Split	90%	
Lumped, One initial		31%
Lumped, Both initials		38%

Here, precision reflects the extent of error due to name variants from 100% splitting; and recall, the extent of error due to grouping of common name elements from 100% lumping.

**Table 3:** The number of operations required for each simulation in Figures 3, 4, 6, and 7 corresponding to the number of name instances eligible for identity change.

	*PubMed*	*USPTO*
Splitting	383,436	26,685
Lumping, One initial	1,997,939	285,689
Lumping, Both initials	1,374,465	179,341

**Figure 3:**
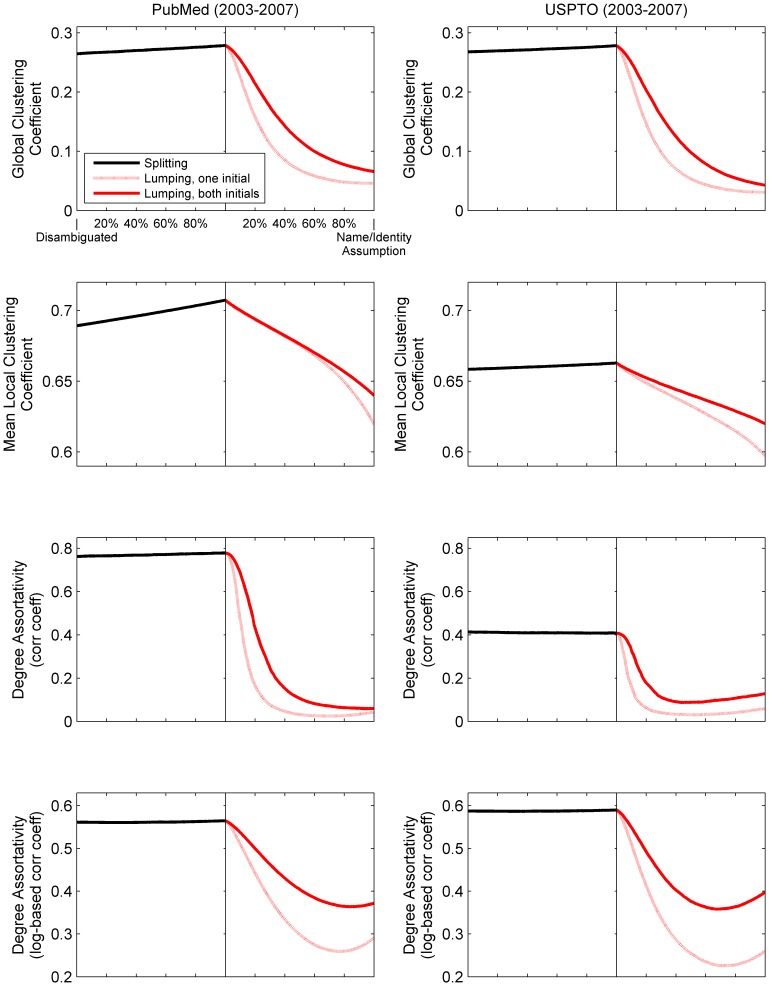
Change in clustering coefficient and degree assortativity given splitting and lumping of PubMed authors (2003–2007) and USPTO inventors (2003–2007). In each subfigure, the x axis denotes the state of completion for splitting and lumping separately; the y axis represents the value of each labeled statistic. Each line segment (differentiated by color and style) plots 100 separate snapshots of the underlying network taken at even intervals for each set of operations. Splitting is based on last name, both initials. See Table 3 for the number of operations required. The global clustering coefficient is due to Equation 1; the mean local clustering coefficient to Equation 2. Degree assortativity is calculated as the correlation coefficient (corr coeff) with linear scaling and, separately, log-based scaling of degree.

**Figure 4:**
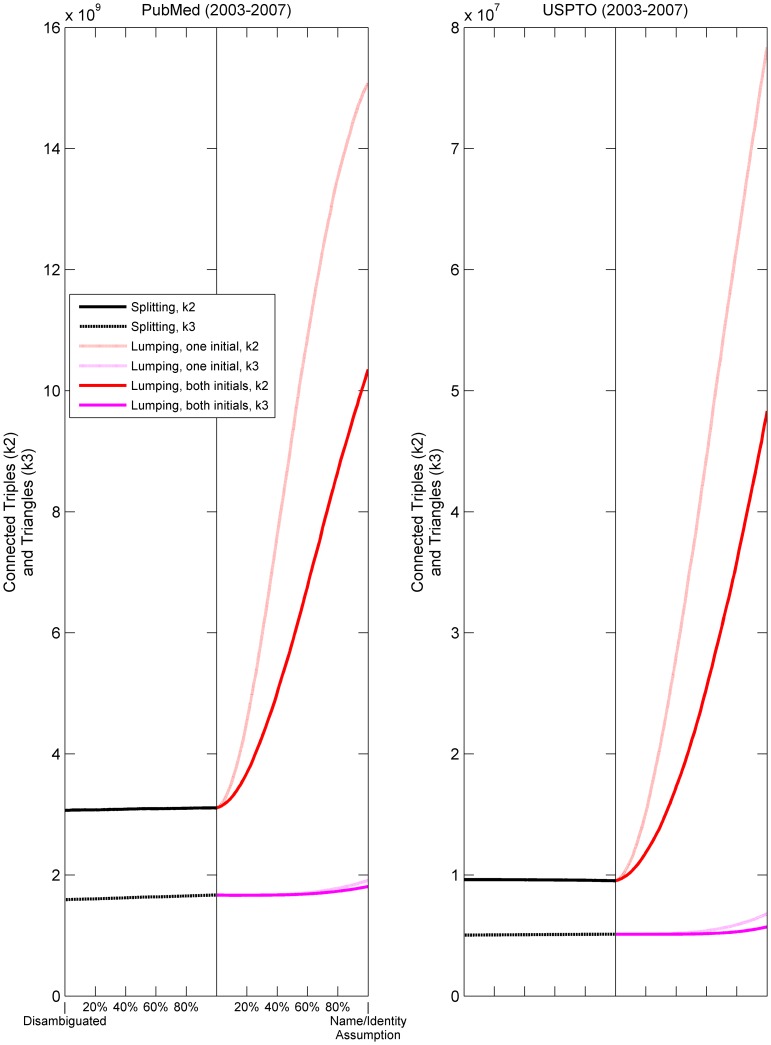
Change in triangles and connected triples given splitting and lumping of PubMed authors (2003–2007) and USPTO inventors (2003–2007). In each subfigure, the x axis denotes the state of completion for splitting and lumping separately; the y axis represents the value of each labeled statistic. Each line segment (differentiated by color and style) plots 100 separate snapshots of the underlying network taken at even intervals for each set of operations. Splitting is based on last name, both initials. See Table 3 for the number of operations required.

Figures 3 and 4 show the extent to which some network measures change as functions of the extent of splitting and lumping, from start to finish (from no splitting or lumping to 100% splitting and lumping). They show that network measures vary considerably, but systematically, and behave differently under splitting and lumping. In Figure 3, for example, the systematic distortion of the global clustering coefficient, *C*, and degree assortativity is particularly dramatic. Intuitively, one might expect *C* to increase with lumping and decrease with splitting. After all, clustering and lumping are both related concepts, colloquially. The fact that the opposite occurs is therefore counterintuitive. The simulations show that for PubMed, splitting increases *C* linearly and only slightly from 0.265 to 0.279, start to finish. For lumping (by last name, both initials), *C* decreases from 0.279 to 0.066, which is the identical value reported for MEDLINE by [37]. Figure 4 shows that this dramatic decrease due to lumping is caused by a dramatic increase in the number of connected triples (the denominator in Equation 1) with little corresponding change in the number of triangles (the numerator in Equation 1). Note that MEDLINE is a subset of PubMed. The use of only one initial makes the effect of lumping even worse. (See dotted lines in Figures 3 and 4.) The change is much less dramatic for the mean local clustering coefficient, in large part because high-degree authors receive the same weight as low-degree authors.

**Figure 5:**
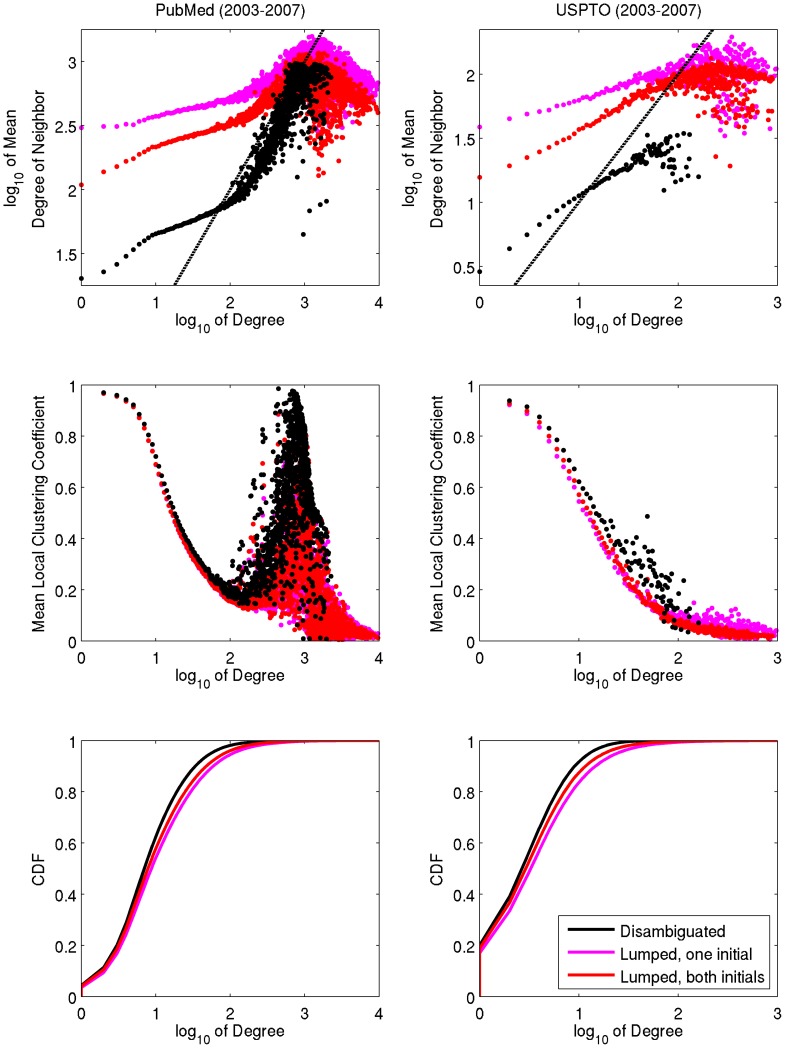
Degree distribution and its relationship with the local clustering coefficient and degree assortativity. Each point represents the average of a set of authors (inventors) with identical degree. The points near the dashed diagonal reflect the influence of hyper-authorship.

Lumping’s dramatic effect on degree assortativity in Figure 3 is explained by Table 4 and Figure 5. In the case of both PubMed and USPTO, covariance between degree and mean degree of neighbors is evident up to a point (a degree of ∼100 for PubMed; ∼30 for USPTO). As the cumulative distribution function shows, most authors (inventors) have degrees below this point, making those above the point “outliers”. These outliers represent (a) authors (inventors) who have indeed collaborated with many other authors (inventors) between 2003 and 2007, (b) lumped individuals, even a few in the disambiguated datasets (such as Lee, Gotoh, Wu, Torres, and Hwang), and (c) for PubMed particularly, the sociological phenomenon of hyper-authorship. Degree assortativity (as expressed using Pearson’s *r*) is sensitive to vertices of high degree; so despite the location of mass in the plots in Figure 5, the outliers (however defined) add sufficient weight to decrease the measure. To test this hypothesis, we selected a subset of authors from PubMed having degree no greater than 100 and papers with no more than 50 authors. Table 4 shows that the removal of high-degree vertices and hyper-authorship papers has little effect on the relative difference between the disambiguated and non-disambiguated networks. After factoring out high-degree vertices and hyper-authorship, lumping still deflates measures of the assortativity and clustering. However, the dramatic effect of lumping is also a direct result of the measures chosen (local vs global clustering, and linear vs. log-based degree assortativity).

**Table 4:** Comparison of different ways of measuring the clustering coefficient and degree assortativity.

Network	Subset	*_C_*	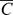	r	r log
**PubMed (2003-2007)**					
Disambiguated	All	0.265	0.689	0.763	0.561
	*_d_*_≤100; *n*≤50_	0.073	0.688	0.164	0.249
Lumped, One initial	All	0.046	0.619	0.043	0.291
	*_d_*_≤100; *n*≤50_	0.018	0.616	0.128	0.194
Lumped, Both initials	All	0.066	0.640	0.060	0.372
	*_d_*_≤100; *n*≤50_	0.020	0.638	0.136	0.208
**USPTO (2003-2007)**					
Disambiguated	All	0.268	0.658	0.414	0.588
Lumped, One initial	All	0.031	0.597	0.059	0.260
Lumped, Both initials	All	0.043	0.620	0.128	0.398

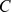
represents the global clustering coefficient; 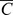, the mean local clustering coefficient; r, degree assortativity (the correlation coefficient of degree); and r log, the correlation coefficient of the log of degree. To eliminate the effects of hyper-authorship, subsets labeled 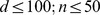
include authors with degree (d) no more than 100 and papers (n) with no more than 50 authors. Subsets labeled “all” exclude nothing.

Splitting has much less effect on network measures than lumping. Because we expect PubMed (and USPTO) to have more shared names than name variants, the magnitude of change due to splitting versus lumping is not surprising. Furthermore, the splitting curves tend to be linear. That is, the distortion of network measures is proportional to the extent of splitting. In contrast, lumping has nonlinear curvature and much greater effect at early stages along the operational continuum. In other words, the distortion of the network measures is *not* proportional to the extent of lumping. The differences between splitting and lumping have at least three possible explanations. First, they may simply reflect fewer splitting than lumping operations, because individuals have fewer name variants than shared names on average. Second, lumping might have a greater domino effect that compounds errors with each lumping operation. Third, lumping may be hitting a floor effect that limits how low a measure can drop.

**Figure 6:**
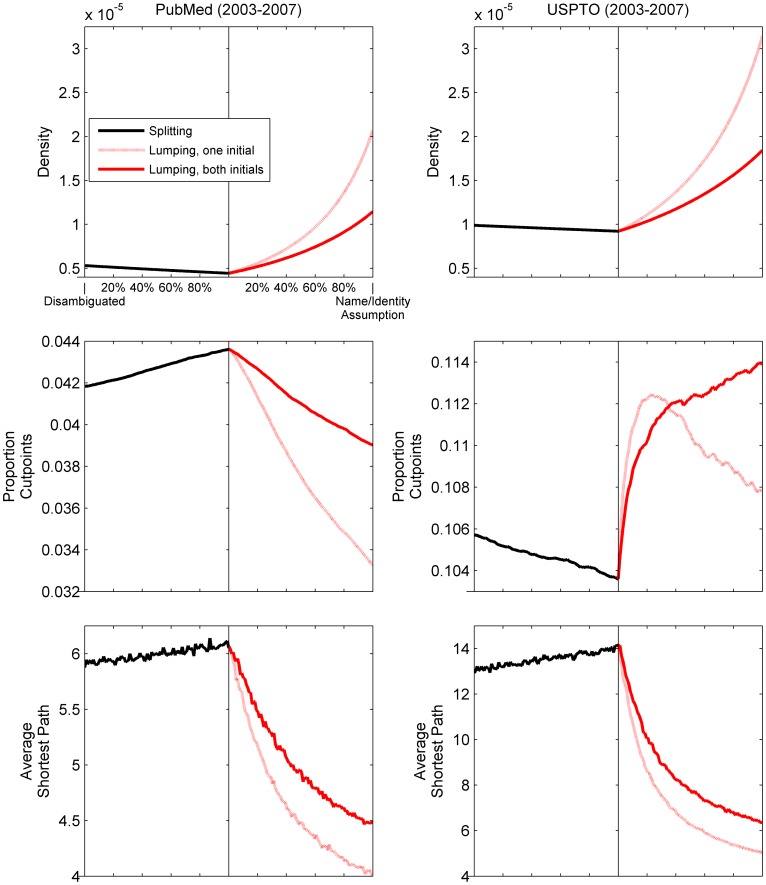
Change in density, the proportion of cutpoints, and average shortest path given splitting and lumping of PubMed authors (2003–2007) and USPTO inventors (2003–2007). In each subfigure, the x axis denotes the state of completion for splitting and lumping separately; the y axis represents the value of each labeled statistic. Each line segment (differentiated by color and style) plots 100 separate snapshots of the underlying network taken at even intervals for each set of operations. Splitting is based on last name, both initials. See Table 3 for the number of operations required.

The proportion of cutpoints to vertices in Figure 6 exhibits opposite reactions (a decrease for PubMed; an increase for USPTO) and, in the case of lumping under USPTO. Although the range of the effect is relatively small (less than one hundredths of a point), this result is unexpected, because the behavior of all other measures is nearly identical, including the relative effect of using one vs. two initials. We expected lumping to create cutpoints, but lumping appears to be creating only local cutpoints. What explains these differences? First, dissimilarities exist in the completeness (perhaps also quality) of the underlying metadata as well as the methods used for disambiguation. For example, the proportion of clusters with missing middle initials from the start of lumping is 63% for PubMed, 48% for USPTO. This implies that more authors are being lumped under the “last name, both initials” name = identity assumption than inventors. Second, the domains of authorship in biomedicine and inventorship broadly may have sociological differences that the tally of cutpoints captures. If this is true, because cutpoints do not exist without biconnected components, the latter warrant further investigation.

**Figure 7:**
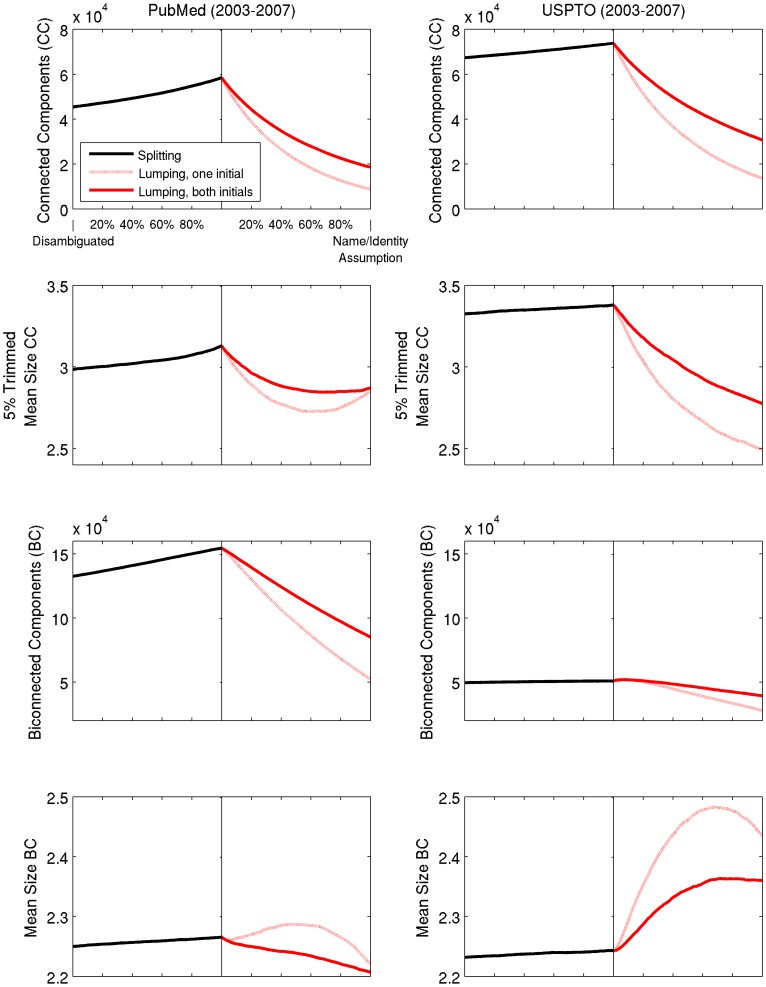
Change in measures of components given splitting and lumping of PubMed authors (2003–2007) and USPTO inventors (2003–2007). In each subfigure, the x axis denotes the state of completion for splitting and lumping separately; the y axis represents the value of each labeled statistic. Each line segment (differentiated by color and style) plots 100 separate snapshots of the underlying network taken at even intervals for each set of operations. Splitting is based on last name, both initials. Differences in the mean size of biconnected components between PubMed and USPTO suggest a cause of the unexpected behavior of cutpoints in Figure 6. See Table 3 for the number of operations required.

Figure 7 shows that the number of biconnected components increases with splitting and decreases with lumping for both PubMed and USPTO. Ostensibly, as the number of biconnected components increases due to splitting, more vertices become cutpoints, maintaining network cohesion. Likewise, as the network contracts due to lumping, cutpoints disappear due to component consolidation (increased component size). PubMed exhibits this behavior; USPTO does not, despite the nearly identical average size of biconnected components for disambiguated versions of both PubMed and USPTO. Something else must be at play.

Table 1 and Figures 6 through 9 provide some clues. USPTO is a much smaller network than PubMed and has a significantly *higher* number of biconnected components (higher by a factor of ∼1.64), a longer average shortest path (more than double for splitting and for the early stages of lumping), and constituent inventors with 3 times fewer collaborators on average. For USPTO, splitting based on name variants alone tends to increase the size of existing biconnected components and decrease the number of cutpoints. Lumping based on the name = identity assumption tends to create more bridges in early stages and thus increases the number of cutpoints. The effect of this behavior is long-lasting for the more selective “both initials” assumption. However, lumping by first initial, being more inclusive and thus prone to greater consolidation of biconnected components, causes a gradual decrease in the number of cutpoints after reaching critical mass at about 20% (Figure 6). The implication here is that the number of biconnected components actually captures a sociological difference between domains.

**Figure 8:**
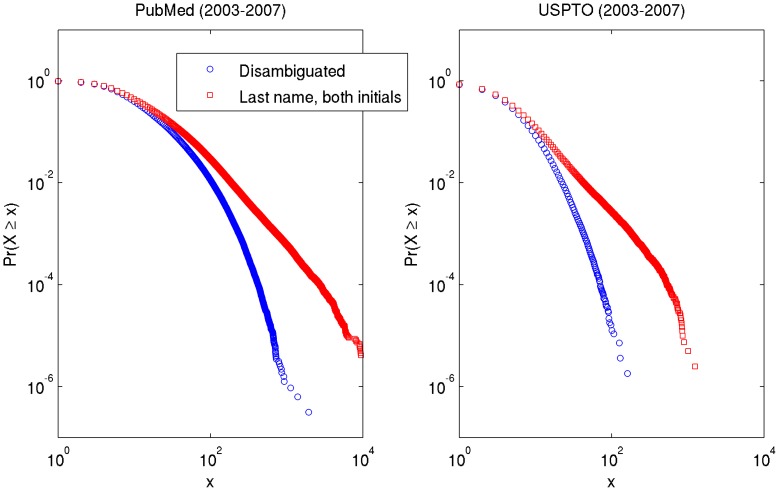
Cumulative distributions of collaborator counts (degree) for PubMed (2003–2007) and USPTO (2003–2007). Note that in both cases, the disambiguated data exhibits much more curvature than for the name = identity assumption.

**Figure 9:**
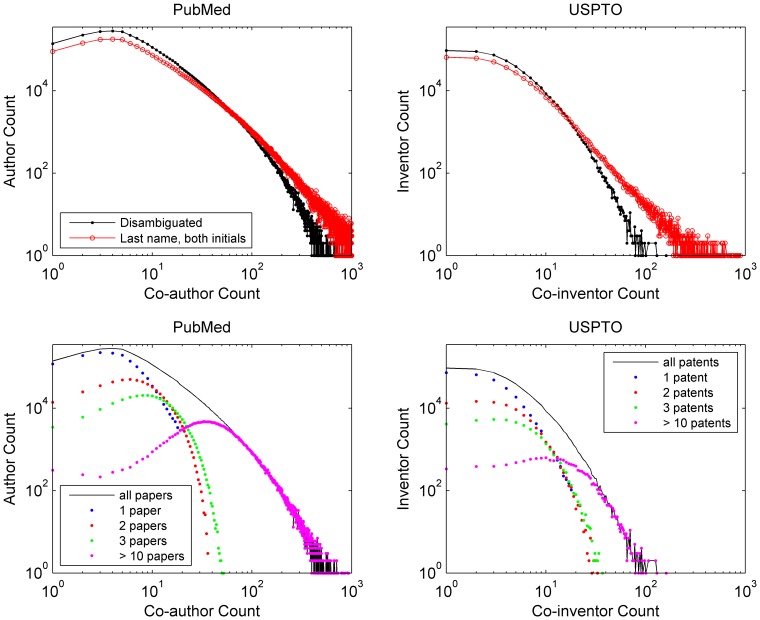
Distributions of collaborator counts (degree) conditioned on paper and patent counts for PubMed (2003–2007) and USPTO (2003–2007). Papers and patents with 20 or more authors or inventors are excluded. Lumping error is visible in the upper row of plots as the name = identity assumption inflates collaborator counts. For PubMed, 280,446 (9%) authors have 4 co-authors over the period; for USPTO, 93,540 (17%) inventors have no co-inventors. For authors with 1 paper, 3 co-authors is the mode; for authors with over 10 papers, 33 co-authors.

Among other measures, density increases dramatically from 0.44×10*^−^* ^5^ to 1.15×10*^−^* ^5^ for lumping with both initials (Figure 6). This result is unenlightening, however. Density is known to increase in networks in which name homonymy is pervasive, such as research groups with Asian affiliations [16]. Moreover, we expect density to be low given the large size of the networks [53].

Collaboration networks such as ones constructed from co-authorships in PubMed and co-inventorships in USPTO are often characterized as scale-free networks. Scale-free networks exhibit a power law degree distribution whereby most vertices have few edges and a small minority of vertices have many edges (making such vertices hubs). When plotted on log-log scale, an empirical degree distribution follows a straight line when it follows a pure power law. While a straight-line fit on a log-log plot is not sufficient evidence of a power law; it is necessary [58], [60], [61]. Both Table 5 and Figure 8 show explicitly that the networks constructed from PubMed and USPTO do not follow a pure power law, with or without disambiguation; rather, the power law is limited to a certain range. Much of the range of the power law disappears with disambiguation. Figure 8 shows that the empirical distributions have at least three components: an initial “hook” (or curve); a line; and a curved tail (or cutoff). (The mixture model in Figure 9 shows this too. Note [6], [62].) The initial hook in the curves probably reflects the fact that most authors represented in the PubMed dataset obtain three to four co-authors in a single instance. Inventors behave similarly; but the hook is narrower, because their norm favors sole or dual attribution. The curved tail probably reflects the finite limits of human capacity (physical and mental). For a moderate number of collaborators, we see a linear trend in the non-disambiguated set, whereas the disambiguated set exhibits some curvature throughout the moderate range (as shown by the narrow range of the linear fit in Figure 8). In other words, the apparent power-law fit becomes an effect of ambiguity, because disambiguation reduces the linear piece of the distribution.

**Table 5:** Basic properties of the PubMed (2003-2007) and USPTO (2003-2007) networks along with power-law fits of their degree distributions.

Network	n	Mean	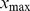	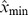	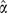	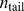
**PubMed (2003–2007)**						
Disambiguated	3,188,865	13.7 (21.7)	1,944	156 (13)	4.3	11,961 (3,275)
Last name, Both initials	2,197,836	20.3 (72.4)	15,647	131 (18)	2.7	42,773 (7,427)
**USPTO (2003–2007)**						
Disambiguated	555,740	3.9 (4.6)	161	26 (3)	4.6	3,993 (1,385)
Last name, Both initials	403,097	5.4 (14.5)	1,247	13 (3)	2.7	35,399 (6,387)

The power law distribution has the form 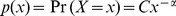, for 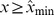, where *x* is degree, *C* is the normalizing constant 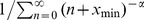; ***n*** is the number of observations; and 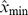 is the estimated lower bound (cutoff) of the scaling region such that 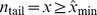. The values here were computed from 1,000 iterations of the non-parametric fitting procedure using the goodness-of-fit approach described by [58]. Because the p-value for each distribution is zero with large 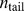 and small 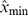, we have strong evidence that none of the distributions fit a power law. (See also [59]).

*Note:* Standard deviation shown in parentheses.

Figure 9 shows co-authorship degree distributions decomposed into mixtures of authors by productivity. The bottom two subfigures show that the curvature of both baselines beyond four collaborators results from increased output (papers published or patents granted) and, perhaps not coincidentally, a larger number of collaborators over time. The curvature may indicate the physical limits of what someone can accomplish in a finite period (over 5 years), the number of people with whom one can collaborate, and/or the number of people with whom one can maintain stable collaborative relationships. (This effect may be seen in the tail of plots in Figure 8 as well.) For example, although [11] did not account for name ambiguity, they found that team size varies by discipline and tends to increase up to a local optimum over time. Generalizing from Figure 9, relatively few authors or inventors collaborate with many other authors or inventors. This is the essence of the power law in collaboration networks: a few individuals are extremely effective in building on their existing social and topical capital, producing a compounding effect.

## Discussion

From analysis of two large networks constructed from PubMed and USPTO, we see dramatic changes in statistical properties when using only name attributes to determine the identities of authors and inventors. These changes indicate that these properties capture topological aspects of the networks that are sensitive to splitting and lumping errors. In Figure 3, for example, degree assortativity increases modestly due to splitting and decreases precipitously due to lumping. Additionally, relatively few lumping operations account for 80% of the changes in both cutpoints and average shortest path. These findings challenge the reasoning expressed by both [2] and [37] that name ambiguity has little effect in large-scale network analyses. It also reinforces the importance of contextual clues (such as collaborators, affiliations, and topics) in establishing identity and affirming attribution. (Note [26], [28]).

*Why does lumping have such a dramatic effect on network measures?* The effect of lumping on network measures has at least two explanations: (a) ambiguity; (b) sensitivity to high-degree vertices (e.g., due hyper-authorship or lumped individuals). First, lumping tends to create high-degree vertices and deflate transitivity measures by artificially creating 2-stars and local cutpoints (not global cutpoints as captured by the cutpoint measure). As lumping increases the number of local biconnected components, it transforms the network into a more hierarchical structure. Second, measures such as the global clustering coefficient and degree assortativity are particularly sensitive to the presence of high-degree vertices. (Kowalski [63] noted that this sensitivity may also be a consequence of measures for which the underlying distribution is assumed to be nearly normal.) The linear scale upon which degree correlation is calculated fails to capture positive degree assortativity among low-degree vertices. High degree vertices are also influential in the global clustering coefficient, not because of its scale, but rather because of its weighting scheme: it counts triples. By averaging or rescaling individual measures (as with the mean local clustering coefficient and log-based degree assortativity, respectively), one can discount the weight some aggregate measures give high-degree vertices. With such discounting, the differences observed with the otherwise degree-sensitive measures become much less dramatic. It should also be noted that hyper-authorship papers create local networks wherein nearly everybody has the same (high) degree and are related transitively. Removing hyper-authorship from a dataset is one possible corrective action; but the utility of the resulting network and the measures used to characterize it depend on the research questions asked.

*When splitting and lumping have opposite effects, why don’t they cancel each other? What explains lumping’s nonlinear effect?* First, lumping is much more prevalent than splitting, because a person has fewer name variants than individuals with whom he or she shares a name, both on average and in the extreme. That is, people with name variants typically have just two but could have a dozen; however, an ambiguous name is typically shared by several people and could be shared by thousands. Second, splitting has a local effect, whereas lumping has a global compounding effect. Splitting happens to one person; and a split personality is likely to collaborate with someone closely related to the original. Lumping involves multiple people and links together wildly different parts of a network. Thus, as the number of lumping operations increases, errors compound, creating a much bigger effect on the network. So, both the number of individuals involved and the compounding effect of lumping ensure that (a) splitting and lumping are not mutually cancelling operations and (b) early and late lumping operations differ in their effect on network measures.

*Can corrective factors (offsets) for network measures overcome the need for disambiguation of named entities altogether?* Measures of large-scale named-entity networks are biased if the networks they characterize have not been disambiguated first. Assuming the disambiguated version of a named-entity network is unknown or unknowable, one could compensate for this bias by using a corrective factor. Due to the linear effect of splitting operations on most measures, a corrective factor for splitting only needs estimates of (a) a per-operation splitting effect of partial disambiguation and (b) the total number of splitting operations. Due to the nonlinear effect of lumping operations, a corrective factor for lumping also requires one to characterize the shape of the curve. The shapes corresponding to the networks studied here are highly nonlinear and depend upon the class of network examined (such as small-world, scale-free, and others based on varying degrees of clustering and assortative mixing, assuming generalization to class is possible; [64]–[66]). The degree of nonlinearity, even if monotonic, indicates that the corrective factor for lumping is likely to be much less accurate than the corrective factor for splitting. Derivation of corrective factors could be fruitful future research.

In our investigation, splitting and lumping had opposite effects on named-entity networks in the limit: splitting increased the global clustering coefficient; lumping decreased it. We observed a similar though more striking and abrupt effect in degree assortativity. The name = identity assumption leads to underestimates of such network measures, and the extent of mischaracterization is probably underappreciated. For example, as both the global clustering coefficient and degree assortativity become smaller, the network they characterize becomes more diffuse and less cohesive. For a collaboration network, the smaller values may indicate an increase in the diversity of opinion, experience, perspective, and potential for innovation (via the strength of weak ties). They may indicate isolationism just as well. For other types of networks, the smaller values may also indicate greater vulnerability to targeted attack due to a larger proportion of non-redundant ties [48]. Dependency on the name = identity assumption means that a network so characterized may appear less resilient and more inhomogeneous than it is in actuality.

While name ambiguity facilitates communication, it is a significant and limiting factor in the analysis of named-entity networks. The diverse state of metadata in bibliographic repositories, for example, ensures that resolving ambiguous names remains a difficulty that only increases with scale. Caution is advised in overinterpreting results where name disambiguation is not assured or for which no account is made.
